# Annotations

**Published:** 1907-05-11

**Authors:** 


### Annotations.

### School Hygiene.

In 1904 the first International Congress of School Hygiene was held at Nuremberg, and those British representatives who attended the meetings in the old town lying under the shadow of the blue Franconian mountains, will remember how hospitably and cordially Nuremberg received them. This year, in August, the second session of the Congress will take place in London, and it promises to be a thoroughly representative international meeting. The good purpose served by such a gathering is obvious. It attracts public attention to the urgent necessity for cultivating healthy conditions in the life of the growing child, and of safeguarding, as much as is possible, the health of the school attending youth. Race deterioration, of which we hear so much nowadays, will be largely prevented if the public is made aware, by means of the published discussions at such conferences, of the vital necessity for hygienic environment in our schools and educational and industrial institutions. We cordially welcome the proposal to hold the Congress in London. A representative committee, of which Sir Lauder Brunton is president, has been formed to deal with the work of the Congress, and subsidiary reception, organising, ladies', and finance committees have also been established. A sum of £3,000 is required for the proper and efficient organisation of the Congress. Towards this sum £1,000 has already been collected and the committee now appeals for further support to enable it to represent in a manner worthy of London, a liberal and generous hospitality.

### A Plea for Oatmeal.

Dr. Chalmers Watson, who has already published numerous interesting studies in connection with dietetic problems, now aspires to place the use of oatmeal as a food on a sound scientific basis. Nowadays, what is termed in Scotland "use and wont" is apt to be looked upon as an insufficient defence even for a well-established and generally-recognised food habit. Justification must be provided by the chemical and physiological laboratory. Upon evidence so obtained Dr. Watson founds a suggestion that a large measure of the food value of oatmeal is due to its capacity to stimulate the activity of the thyroid gland. After feeding a number of young rats for four to eight weeks on a diet of uncooked oatmeal and water, an autopsy revealed in each instance considerable enlargement of the thyroid, together with evidences of increased glandular activity. The observation was made all the more striking by the absence of any such changes in a control series of rats who had been fed on a bread-and-milk diet. The suggestion now is that it is by stimulation of the thyroid that porridge produces the excellent results which it can claim as a food for children. Turning to the practical side, Dr. Watson makes one or two detailed proposals for the use of oatmeal. He approves of its use at breakfast in the form of porridge and milk, and advises that the meal shall be completed by a glass of milk and some bread and butter, and shall not include bacon or any other form of meat. If meat is introduced it tends to induce a distaste for the less appetising porridge, which sooner or later, therefore, gets neglected. Rats, equally with children, it seems, display this same dietetic perversity; they will not eat oatmeal or bread when meat is available. As porridge does not encourage the use of the muscles of mastication children should also receive a supply of crusted bread, rusks, etc., and these should be taken "dry" and not washed down with fluid. Lastly, it is necessary to recognise that some children—indeed, the same is true of some adults—cannot digest oatmeal. Food value and digestibility are not one and the same thing, and the public ignorance of this fact is responsible for many misconceptions.

### The Pharmaceutical Society.

The cultivation and development of the art of pharmacy are matters of considerable interest to the medical profession, and it is therefore gratifying to learn from the current Annual Report of the Pharmaceutical Society that in many respects the present position of affairs is a satisfactory one. The Society's school continues to maintain its acknowledged position, and the value of the academic and practical training it provides is shown by the large numbers of students which each year are attracted to its curriculum; we have the pleasure to congratulate the Dean of the School, Professor A. W. Crossley, on his recent election to the Fellowship of the Royal Society. That there is need for extension and improvement of the general level of pharmaceutical education in the country generally is evident from the large percentage of rejections in the Society's examinations. Thus of 1,165 candidates who entered for the "Minor" examination—that is, the examination which has to be passed in order to gain the legal right to use the term "Chemist and Druggist"—764, or a percentage of 65.58, failed to satisfy the examiners. It is to be noted, too, that the greater part of the failures occurred in the practical part of the examination. All this is the repetition of the experience of previous years, and it carries the inevitable conclusion that the practical opportunities offered to the majority of the candidates must be of a very limited description. It is to be feared that no substantial improvement is to be expected until a compulsory curriculum is enforced as a necessary preliminary to the qualifying examination. For the "Major" examination, which confers the title "Pharmaceutical Chemist," there were 79 candidates, and of these 37 were successful. The Museum, the Library, and the *Pharmaceutical Journal* are other activities of the Society, and from all of these the reports continue to indicate a high level of attainment. The following have been elected Honorary Members of the Society: Sir Alex. Pedler, Professors Eugène Collin, Emil Fischer, R. Meldola, and Dr. Dixon Mann.

